# Enhanced ocean oxygenation during Cenozoic warm periods

**DOI:** 10.1038/s41586-022-05017-0

**Published:** 2022-08-31

**Authors:** Alexandra Auderset, Simone Moretti, Björn Taphorn, Pia-Rebecca Ebner, Emma Kast, Xingchen T. Wang, Ralf Schiebel, Daniel M. Sigman, Gerald H. Haug, Alfredo Martínez-García

**Affiliations:** 1 Climate Geochemistry Department, Max Planck Institute for Chemistry, Mainz, Germany; 2 Department of Earth Sciences, ETH Zurich, Zurich, Switzerland; 3 Department of Geosciences, Princeton University, Princeton, NJ USA; 4 Department of Earth Sciences, University of Cambridge, Cambridge, UK; 5 Department of Earth and Environmental Sciences, Boston College, Chestnut Hill, MA USA

**Keywords:** Biogeochemistry, Palaeoceanography, Palaeoclimate

## Abstract

Dissolved oxygen (O_2_) is essential for most ocean ecosystems, fuelling organisms’ respiration and facilitating the cycling of carbon and nutrients. Oxygen measurements have been interpreted to indicate that the ocean’s oxygen-deficient zones (ODZs) are expanding under global warming^1,2^. However, models provide an unclear picture of future ODZ change in both the near term and the long term^3–6^. The paleoclimate record can help explore the possible range of ODZ changes in warmer-than-modern periods. Here we use foraminifera-bound nitrogen (N) isotopes to show that water-column denitrification in the eastern tropical North Pacific was greatly reduced during the Middle Miocene Climatic Optimum (MMCO) and the Early Eocene Climatic Optimum (EECO). Because denitrification is restricted to oxygen-poor waters, our results indicate that, in these two Cenozoic periods of sustained warmth, ODZs were contracted, not expanded. ODZ contraction may have arisen from a decrease in upwelling-fuelled biological productivity in the tropical Pacific, which would have reduced oxygen demand in the subsurface. Alternatively, invigoration of deep-water ventilation by the Southern Ocean may have weakened the ocean’s ‘biological carbon pump’, which would have increased deep-ocean oxygen. The mechanism at play would have determined whether the ODZ contractions occurred in step with the warming or took centuries or millennia to develop. Thus, although our results from the Cenozoic do not necessarily apply to the near-term future, they might imply that global warming may eventually cause ODZ contraction.

## Abstract (web summary)

By using foraminifera-bound nitrogen isotopes, it is shown that, during two warm periods of the Cenozoic, oxygen-deficient zones contracted rather than expanded, suggesting that global warming may not necessarily lead to increased oceanic anoxia.

## Main

Observations indicate that oxygen concentrations have been decreasing in coastal and open-ocean waters over the past five decades, substantially expanding the volume of the ocean’s ODZs^1,2^. The global deoxygenation trend has been attributed to the decreased oxygen solubility and enhanced upper-ocean stratification expected from global warming, with predictions that global oxygen concentration will continue to decrease over the next decades and centuries, affecting marine ecosystems^7^. However, there is controversy as to the future of the ODZs in particular, with predictions of both expansion and contraction^3–6^.

Earth’s climate has evolved in response to tectonic and orbital forcing over the Cenozoic era, the past 66 million years (Myr). Overall, atmospheric CO_2_ concentrations have decreased and global climate has cooled^8^. Against this background of Cenozoic cooling, there were two distinct periods of prolonged warm climate: the MMCO, about 6 °C warmer than today, and the EECO, about 13 °C warmer than today^9^. The MMCO and the EECO were characterized by higher CO_2_ (around 550 ppm and 1,650 ppm, respectively) and reduced or no continental ice^10–12^. These two climate optima present an opportunity to investigate the response of ocean oxygen concentrations to prolonged warmth^13^.

The ratio of N isotopes in the ocean is sensitive to oxygen deficiency, or ‘suboxia’ (a dissolved O_2_ concentration of less than approximately 5 µmol kg^−1^, based on standard measurements). Bacterial reduction of nitrate to N_2_ (‘denitrification’) requires suboxia and discriminates against ^15^N. Consequently, denitrification in the ODZs of the ocean water column causes an elevation in the ^15^N/^14^N ratio, or δ^15^N, of nitrate in the regions of the ODZs^14^, also increasing the nitrate δ^15^N of the global ocean^15^ (δ^15^N = ((^15^N/^14^N)_sample_/(^15^N/^14^N)_atmN2_ − 1)) × 1,000‰). The isotopic signal of ODZ denitrification is incorporated into newly formed biomass in the surface ocean, the by-products of which accumulate in deep-ocean sediments. Thus, the N isotopes provide a tool for reconstructing past changes in ODZ-hosted denitrification^16^. Organic matter that is native to and trapped within the mineral matrix of planktic foraminifer shells (foraminifer-bound organic matter) has been developed as an archive of the N isotopic history of surface ocean productivity^17^. Foraminifer-bound organic matter is isolated from the sedimentary environment by the mineral matrix, preserving the original unaltered N isotopic signal even over millions of years^18^. In areas of complete nitrate consumption, foraminifer-bound δ^15^N records the δ^15^N of the nitrate in the underlying pycnocline^19^. Pycnocline nitrate δ^15^N bears regional signals of water-column denitrification and of N_2_ fixation, the dominant source of biologically available (that is, ‘fixed’) N to the ocean^20^. Pycnocline nitrate δ^15^N will also vary with the δ^15^N of mean ocean nitrate. Mean ocean nitrate δ^15^N, in turn, is largely controlled by the relative proportions of denitrification occurring in the water column versus the sediments^15^.

## N isotope and temperature reconstructions

**Fig. 1:**
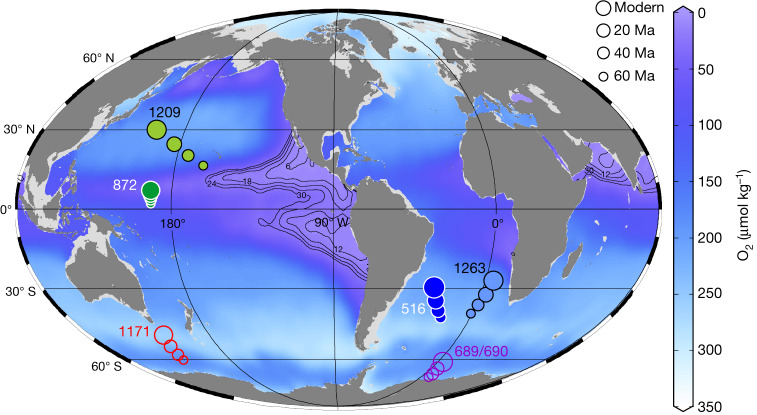
Sites from which core data are reported or discussed, plotted over modern dissolved O_2_ concentrations at 350 m water depth. Tectonically driven changes in site locations are shown with symbol size (see legend). Filled circles indicate cores with FB-δ^15^N data, from this study (872/516) and from Kast et al.^18^ (1209/1263), and open circles indicate cores with foraminiferal δ^18^O data discussed in the text^37,41^ (and references therein). Dissolved O_2_ concentrations (in μmol kg^−1^) are shown in colour.

**Fig. 2:**
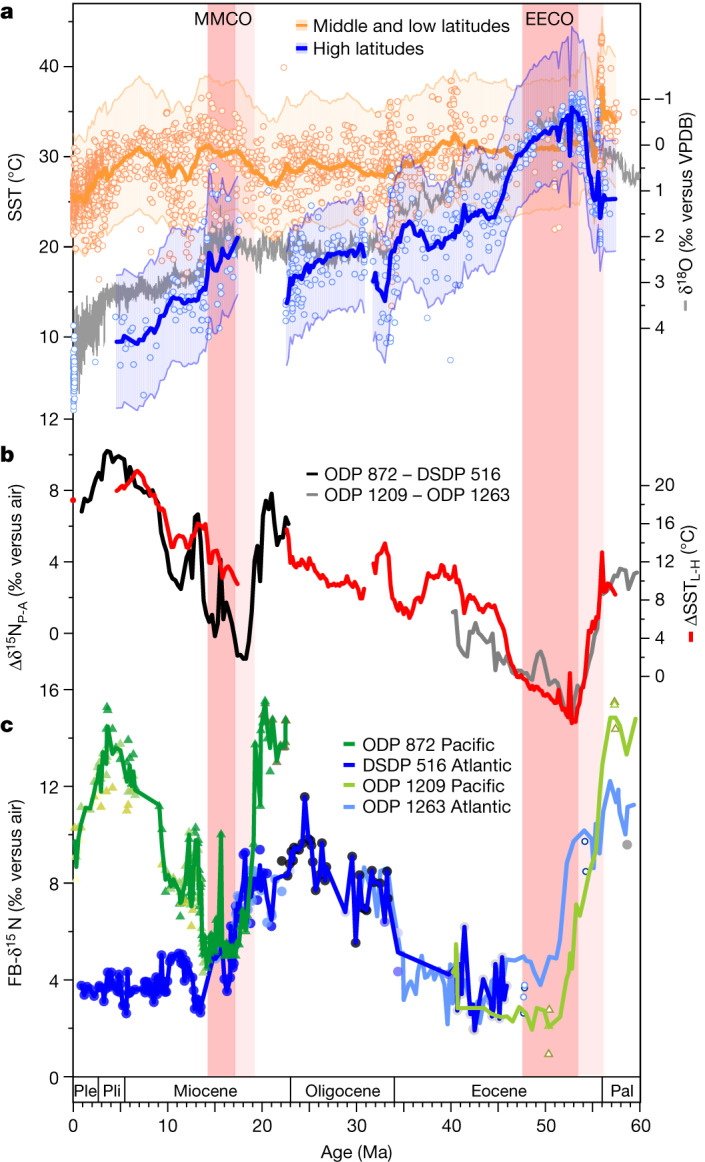
Evolution of FB-δ^15^N and climate over the Cenozoic. **a**, Compilation of new and previously published SST data based on the TEX_86_ palaeothermometer, divided into high latitudes (above 50° N/S) and low/middle latitudes (0–40° N/S), with LOESS smoothing (factor 0.02) and 90% confidence interval (see Methods and Supplementary Data 2). TEX_86_ temperature estimates are based on the BAYSPAR calibration (see Methods). The grey line shows the benthic foraminiferal oxygen isotope ratio (δ^18^O) compilation by Westerhold et al.^8^. **b**, Δδ^15^N_P-A_ is the FB-δ^15^N difference between Pacific ODP Site 872 and Atlantic DSDP Site 516 (black line) or between Pacific ODP Site 1209 and Atlantic ODP Site 1263 (grey line). ΔSST_L-H_ (red line) is the SST difference between low/middle latitudes and high latitudes (from **a**; the propagated 90% confidence interval for ΔSST_L-H_ is shown in Extended Data Fig. 4b). **c**, Average of species-specific FB-δ^15^N from ODP Site 872 (green) and DSDP Site 516 (blue), together with mixed-taxa FB-δ^15^N from ODP Site 1209 (light green) and ODP Site 1263 (light blue), calculated as the average of the two size fractions^18^. Triangles and circles of different colours indicate individual species-specific or genus-specific FB-δ^15^N in Site 872/1209 and Site 516/1263, respectively (see Extended Data Fig. 1 for details). Light pink bars indicate the first signs of global warming into the EECO and the MMCO, and darker bars indicate the main warm intervals, based on the benthic foraminifer δ^18^O (**a**). Ple, Pleistocene; Pli, Pliocene; Pal, Palaeocene.

**Extended Data Fig. 1:**
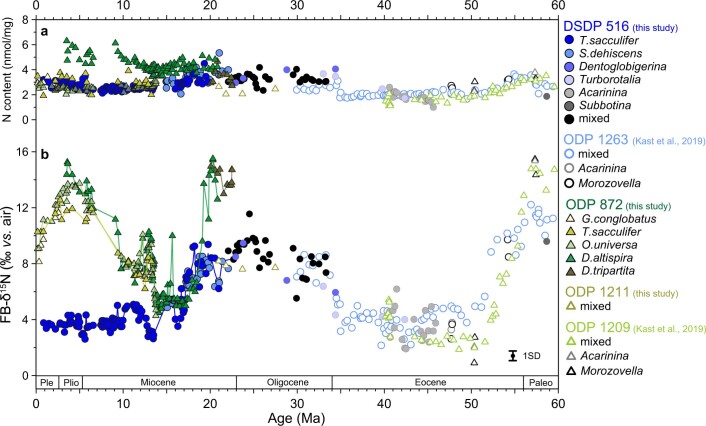
Cenozoic foraminifer-bound nitrogen isotope and nitrogen content data compilation, with sites and taxa indicated. Foraminifer-bound N content (**a**) and FB-δ^15^N (**b**) for species-specific analyses in DSDP Site 516, ODP Site 872 and ODP Site 1211, complemented by data from mixed taxa and specific genera by Kast et al.^18^ for ODP Sites 1263 and 1209. Our compilation is of putatively symbiotic taxa or coarse fractions dominated by symbiotic taxa, avoiding the need to correct for the typical offset between symbiotic and symbiont-barren species^19,89^. Cross-plots between δ^15^N and N content show no correlation.

In this study, we measure the δ^15^N of foraminifer-bound organic matter (FB-δ^15^N) to investigate changes in the marine N cycle over the Cenozoic. We present new species-specific FB-δ^15^N data since the Miocene, as well as genus-specific measurements across the early Miocene, Oligocene and late Eocene epochs from two sediment cores, one located in the Pacific (ODP Site 872) and the other in the Atlantic (DSDP Site 516) (Fig. 1). These data are compiled with previously published mixed-taxa and genus-specific FB-δ^15^N data from the Eocene and Palaeocene epochs at Pacific ODP Site 1209 and Atlantic ODP Site 1263, respectively (Fig. 1), with the merged datasets covering much of the Cenozoic (Fig. 2c and Extended Data Fig. 1).

**Extended Data Fig. 2:**
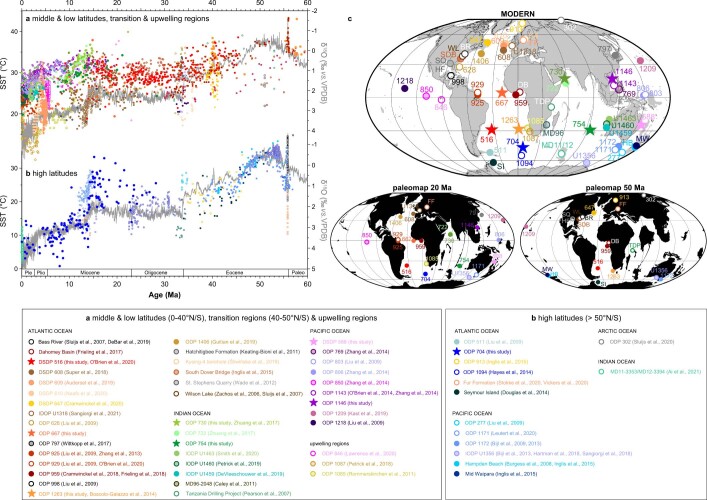
Cenozoic TEX_86 Bayspar_-derived SST compilation. SSTs are categorized in middle and low latitudes with transition and upwelling regions (**a**) and high latitudes (**b**). Grey line indicates benthic oxygen isotope compilation by Westerhold et al.^8^. **c**, Maps with core locations: modern map shows current locations of core sites. Palaeomap at 20 Ma with palaeorecords spanning 10–30 Ma and palaeomap at 50 Ma with palaeorecords spanning 40–60 Ma. Maps are adapted from the Ocean Drilling Stratigraphic Network (ODSN) Paleomap project (http://www.odsn.de/odsn/services/paleomap/paleomap.html, accessed May 2022). Palaeolatitudes for core sites are calculated after van Hinsbergen et al.^52^.

**Extended Data Fig. 3:**
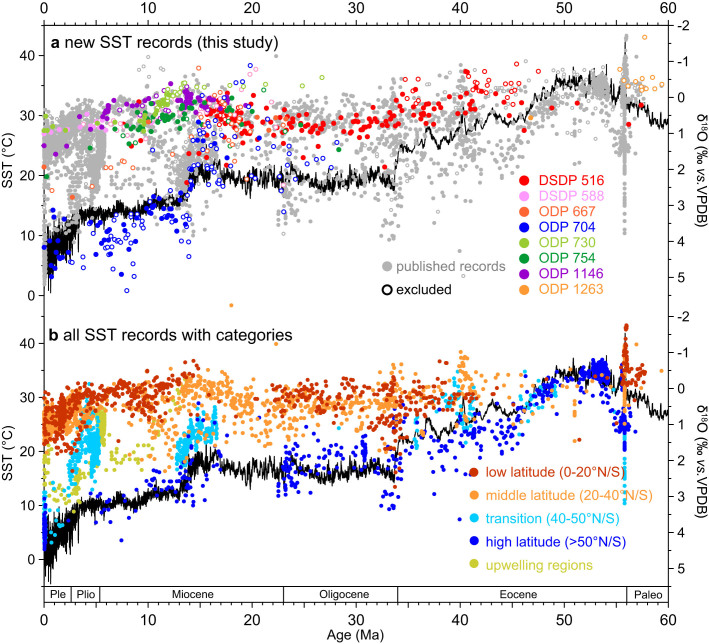
Cenozoic TEX_86 Bayspar_-derived SST compilation, categorized on the basis of latitude. **a**, TEX_86 Bayspar_ -derived SST data generated in this study are in red (DSDP Site 516), pink (DSDP Site 588), dark orange (ODP Site 667), blue (ODP Site 704), light green (ODP Site 730), green (ODP Site 754), purple (ODP Site 1146) and light orange (ODP Site 1263). In grey are published SST records from our compilation (Supplementary Data 2). Open dots of different colours indicate samples that have been excluded due to anomalous GDGTs distributions (see Methods). **b**, TEX_86 Bayspar_-derived SST records categorized in low (0–20° N/S, red) and middle (20–40° N/S, orange) latitudes, transition regions (40–50° N/S, light blue), high latitudes (>50° N/S, blue) and upwelling regions (green). Black line indicates benthic oxygen isotope compilation^8^.

**Extended Data Fig. 4:**
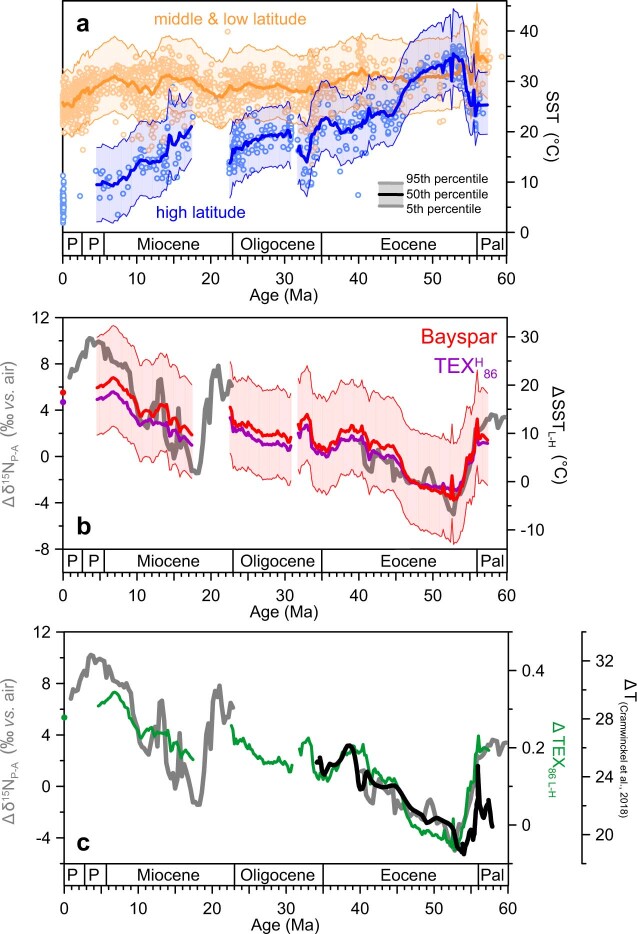
Comparisons and confidence intervals for TEX_86_-derived SST reconstructions. **a**, TEX_86 Bayspar_-derived SST for middle and low (orange) and high (blue) latitudes datasets with corresponding 90% confidence interval. **b**, Comparison between δ^15^N-gradient (Δδ^15^N_P-A_) and latitudinal SST-gradient (ΔSST_L-H_) calculations, using two SST calibrations: SST_Bayspar_ after Tierney and Tingley^138^ (with the propagated 90% confidence interval from **a**) and SST_TEXH_ after Kim et al.^137^. **c** Latitudinal TEX_86_-gradient (ΔTEX_86 L-H_) in comparison with the latitudinal SST gradient proposed by Cramwinckel et al.^99^. (Δ*T* = SST_TEXH86_ − SST_δ18O_, black line). Note that ΔTEX_86 L-H_ does not depend on the calibration used.

In addition, we have generated a TEX_86_-based^21^ sea surface temperature (SST) compilation that includes new measurements as well as previously published data (Fig. 2a and Extended Data Figs. 2 and 3). The compilation shows the thermal evolution of the upper ocean at low/middle and high latitudes. The SST compilation allows us to reconstruct the low-to-high-latitude SST gradient (ΔSST_L-H_) using a single temperature proxy (Fig. 2b and Extended Data Figs. 2–4). ΔSST_L-H_ may be a particularly sensitive indicator of past climate change, and it provides constraints on the circulation of the atmosphere and ocean.

The new data show that Atlantic and Pacific FB-δ^15^N values were elevated with respect to the present during the early Miocene but decreased sharply in the two basins from 19 to 16 million years ago (Ma), with a greater decline in the Pacific (of around 11‰) than in the Atlantic (about 6‰) (Fig. 2c). FB-δ^15^N reached a minimum at both sites during the MMCO. FB-δ^15^N then increased during the subsequent cooling phase (from 14 Ma to 10 Ma), known as the Middle Miocene Climate Transition, again with a greater change in the Pacific than in the Atlantic. Finally, during the late Miocene (from 10 Ma to 5 Ma), the records decouple: the Pacific record shows a strong FB-δ^15^N increase followed by a decline at 3 Ma, whereas the Atlantic record first shows a small decrease and then remains stable until the present.

The compiled data indicate convergent behaviour for the MMCO and the EECO. Both climate optima were characterized by lower Pacific FB-δ^15^N and a decrease in the FB-δ^15^N difference between the Pacific and the Atlantic (Δδ^15^N_P-A_; Fig. 2b,c). Moreover, both climate optima were associated with strong warming of the high-latitude ocean that reduced the meridional SST gradient (Fig. 2a,b). Δδ^15^N_P-A_ and ΔSST_L-H_ are correlated not only during the warming phases but also more broadly through the Cenozoic (Fig. 2b).

## Reduced suboxia during warm periods

The coupled declines in δ^15^N and Δδ^15^N_P-A_ during the EECO and MMCO are best explained by reductions in water-column denitrification, which today occurs mostly in the eastern tropical Pacific ODZs (Fig. 1). A decline in water-column denitrification, lacking a comparable decline in benthic denitrification, would have lowered the δ^15^N of mean ocean nitrate^15^ and, thus, the δ^15^N in both records. At the same time, if suboxia and water-column denitrification have persistently been concentrated in the Pacific, a decline in water-column denitrification would reduce the elevation of tropical Pacific nitrate δ^15^N relative to the rest of the ocean, lowering Δδ^15^N_P-A_ as observed. Thus, contrary to widely held expectations^22^, our FB-δ^15^N data indicate a reduction, not an enhancement, of the ODZs during the warmest intervals of the Cenozoic.

Both FB-δ^15^N and Δδ^15^N_P-A_ decline sharply at the onset of the EECO. The effect of the Asian–Indian collision on outflow from the Tethys Sea has been proposed as important to this change, through its effect on the temperature of the ocean’s pycnocline^18^. A similar mechanism might be offered for the reconstructed denitrification decline at the onset of the MMCO, when the proto-Mediterranean underwent greater isolation from the Indian Ocean^23^. However, this Tethys-outflow-focused interpretation does not explain the subsequent increases in Pacific water-column denitrification indicated by the increase in Δδ^15^N_P-A_ after each of these warm periods (Fig. 2b).

Other notable tectonic changes roughly overlap with the end of the MMCO. These include the widening of the Drake Passage at 15 Ma after a 10-Myr period of narrowing^24^ and constrictions of the Indonesian Seaway starting at 25–17 Ma (ref. ^25^) and of the Central American Seaway (CAS) starting around 9.2 Ma (ref. ^26^). In a model experiment, CAS closure has been observed to increase regenerated CO_2_ storage in the Pacific interior^27^, presumably enhancing suboxia in the eastern Pacific. This and other effects of the CAS closure may help to explain the increases in Pacific FB-δ^15^N and Δδ^15^N_P-A_ as well as the relative stability of South Atlantic FB-δ^15^N after 9.2 Ma (Fig. 2b,c). However, there is no evidence for constriction of the CAS until after the MMCO had ended^26^. Moreover, even if CAS closure began earlier, it would not explain the decline in Pacific FB-δ^15^N and Δδ^15^N_P-A_ into the MMCO. Finally, CAS closure would not have been relevant for the EECO-associated changes.

**Fig. 3:**
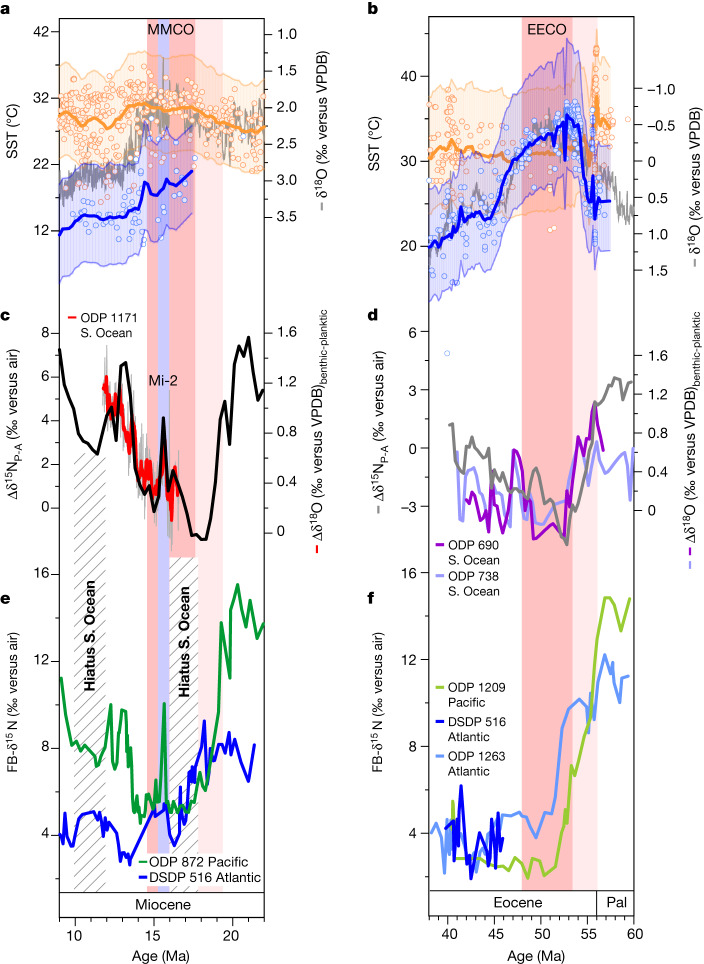
Focus on the two main Cenozoic warm periods, with additional data pertaining to the Southern Ocean. The MMCO is on the left and the EECO is on the right. **a**,**b**, SST compilation along with the benthic δ^18^O compilation^8^, as in Fig. 2. These data indicate, during the climate optima, reductions in the temperature difference between low-latitude and high-latitude surface waters. **c**,**d**, FB-δ^15^N difference between Pacific and Atlantic core sites (Δδ^15^N_P-A_), as in Fig. 2. In **c** and **d**, other lines indicate running averages of the δ^18^O difference between benthic and surface-dwelling planktic foraminifers (Δδ^18^O) in the Southern Ocean (**c**, ODP Site 1171, red line^37,38^; **d**, ODP Sites 690 and 738, purple lines (ref. ^41^ and references therein)). These data suggest a weaker vertical density gradient in the Southern Ocean during the climate optima, consistent with stronger ventilation of the deep ocean from the Southern Ocean surface. **e**,**f**, FB-δ^15^N records for these time intervals. In **e**, the lines indicate the average records of species-specific FB-δ^15^N from ODP Site 872 (green) and DSDP Site 516 (blue). Hatched bars indicate sedimentation hiatuses in the Southern Ocean, which have been attributed to stronger deep-water circulation^39^. In **f**, the lines indicate the average records of mixed-taxa FB-δ^15^N in Site 1209 (light green) and Site 1263 (light blue)^18^, as in Fig. 2. New species-specific FB-δ^15^N data from Site 516 are in dark blue. Pal, Palaeocene; S. Ocean, Southern Ocean.

It is possible that several seaway and/or basin geometry changes conspired to yield the observed MMCO and EECO minima in δ^15^N_P_, δ^15^N_A_ and Δδ^15^N_P-A_. However, the δ^15^N changes that define the minimum at the MMCO are abrupt relative to tectonic changes, and they coincide with the ΔSST_L-H_ changes (Fig. 3). Moreover, within the MMCO, there is an abrupt δ^15^N increase at Site 872, coinciding with the Mi-2 glaciation at 15.9 Ma (Fig. 3c), a cooling event without any known connection to tectonics^28^. These observations argue that the denitrification declines of the MMCO and the EECO are dominantly a common response to the warm conditions of these otherwise distinct periods.

Global ocean nitrate δ^15^N is strongly modulated by the ratio of sedimentary to water-column denitrification, with a higher ratio yielding a lower δ^15^N (ref. ^15^). With warming, the loss of land ice and other processes might cause a sea level rise^12^, encouraging sedimentary denitrification^29^. Such a change might be argued to contribute to the low δ^15^N of the MMCO and the EECO. However, a sea-level-driven change in sedimentary denitrification would produce similar δ^15^N declines in the Atlantic and Pacific basins. Therefore, the larger decline from extremely high δ^15^N at Sites 872 and 1209 cannot be explained solely by enhanced sedimentary denitrification; rather, a reduction in water-column denitrification is required^18^.

## Climate-driven ODZ contraction

Climate models point to several processes that work to shrink the ODZs under global warming, offering potential explanations for our finding of reduced water-column denitrification during the MMCO and EECO. First, a decrease in upwelling-supported biological export production in the equatorial and tropical Pacific would reduce oxygen demand in the subsurface^4,5,30^. Second, circulation changes in the ocean interior, such as enhanced Southern Ocean deep-water formation, may increase oxygen supply^3,6^. Our TEX_86_-based reconstructions of SST and ΔSST_L-H_ are consistent with either mechanism (Fig. 2a,b). On the one hand, the higher global temperatures and weaker ΔSST_L-H_ during the MMCO and the EECO should have weakened the tropical atmospheric circulation^31^, which would have decreased eastern equatorial Pacific upwelling^4^. On the other hand, enhanced deep-water formation in high-latitude regions such as the Southern Ocean may have transported more heat from low to high latitudes, further weakening ΔSST_L-H_ (ref. ^32^).

During the MMCO, a reconstruction of equatorial Pacific hydrography indicates a deepening and flattening of the equatorial Pacific thermocline^33^, which should have caused a reduction in the supply of nutrients to the surface. These findings are consistent with reduced low-latitude export production and, thus, reduced oxygen demand as the cause of ODZ contraction (Fig. 2b). Moreover, a model study^5^ suggests that global warming decreases the nutrient content of the pycnocline waters ventilating the tropics, reducing tropical export production and causing the ODZs to contract. However, evidence for changes in tropical export production across the MMCO and the EECO is scarce and ambiguous^34^. Thus, although a tropical-productivity-related explanation is plausible, it remains unconfirmed.

Open-ocean ODZs occur within the pycnocline, which spans approximately 150–1,200 m depth. Accordingly, the ODZs are undoubtedly sensitive to the biogeochemical conditions and circulation at these ‘intermediate’ depths^4,5^. However, ODZs are also affected by the underlying deep ocean, which contribute water to them through vertical mixing and upwelling, especially near the equator and on the eastern sides of the ocean basins^30^. In the global-warming simulations that predict ocean reoxygenation on centennial and longer timescales, this reoxygenation results from more vigorous ventilation of the deep ocean, driven by enhanced deep convection in the Southern Ocean^3,6^.

The oxygen concentration of the deep ocean (and of the ocean interior as a whole) is tightly linked to the ocean’s ‘biological carbon pump’, the storage of respiratory CO_2_ in deep waters caused by the sinking and subsequent remineralization of organic matter produced in surface waters. A stronger biological pump is associated with more ocean CO_2_ storage, lower atmospheric CO_2_, and lower oxygen concentrations in the deep ocean^35^. The upper limit of the ocean’s biological pump is set by the global mean nutrient (phosphate or nitrate) concentration of the ocean. However, the modern biological pump is weaker than this limit, largely because of ventilation of the deep ocean by the Southern Ocean^36^, which tends to fill the ocean interior with unused nutrients, reducing oxygen consumption in the ocean interior due to the ‘missed opportunity’ for nutrient-fuelled biological productivity at the Southern Ocean surface. Therefore, the decline in water-column denitrification during the MMCO and the EECO may have been driven by stronger Southern Ocean ventilation of the deep ocean.

This possibility is consistent with other observations. During the MMCO, high-resolution Southern Ocean foraminifer oxygen isotope data from ODP Site 1171 (refs. ^37,38^) (with a palaeolatitude of about 55° S) show a lower planktic–benthic difference during the MMCO and an increase after the event, coinciding with the increase in Δδ^15^N_P-A_ (Fig. 3c). These data suggest a reduction in the density gradient between the deep ocean and the Southern Ocean surface, consistent with an increase in deep overturning and a simultaneous weakening of the global biological pump. The records also suggest an episode of stronger Southern Ocean density stratification (high Δδ^18^O_benthic-planktic_), coinciding with the increase in water-column denitrification (high Δδ^15^N_P-A_) during the Mi-2 glaciation at about 15.9 Ma (Fig. 3c). Finally, during the MMCO as well as a pause in the following cooling, the most poleward Southern Ocean sediment cores (ODP Sites 689 and 690) contain hiatuses in sedimentation. These hiatuses have been linked to erosional events triggered by the strengthening of deep-water circulation^39^, further supporting acceleration of deep-ocean ventilation as a cause of the decline in Pacific suboxia (Fig. 3c,e).

During the EECO as in the MMCO, oxygen isotope gradients between planktic and benthic foraminifers decrease substantially in the high-latitude Southern Ocean^40^ (Fig. 3d), again consistent with an increase in deep overturning that coincided with ODZ contraction. In addition, a global ocean reconstruction of the vertical carbon isotope gradient indicates a weaker global biological pump during the EECO^41^. These observations support the possibility that enhanced Southern Ocean overturning contributed to the ODZ contractions of both the MMCO and the EECO. Although our focus has been on Southern Ocean overturning, the North Pacific also shows signs of deep-water formation during warm periods of the Cenozoic^42,43^, a strengthening in which may have had similar effects on the biological pump and ocean oxygen.

A decrease in the efficiency of the ocean’s biological carbon pump would have worked to increase atmospheric CO_2_ during the MMCO and the EECO. This feedback, albeit with presumed buffering by the ‘weathering thermostat’ on the million-year timescale^44^, may have amplified these warm climate events. Palaeobiogeochemical data suggest that the modern Southern Ocean’s ventilation of the deep ocean was reduced during the late Pleistocene ice ages^45^, providing an explanation for the lower oxygen concentration of the ice age deep ocean compared with interglacials and the warm Pliocene^43,46–48^. This interpretation of the Pleistocene glacial cycles implies a climate sensitivity for Southern Ocean ventilation of the deep ocean that is consistent with our observations from the EECO and the MMCO, which suggest that warming tends to induce an increase in ocean-interior oxygen concentrations.

## Implications for future climate

Our measurements indicate that, in past periods of prolonged warmth, ocean suboxia was reduced, not expanded. The possible mechanisms for this change include both short-term and long-term processes^3–6^, leaving uncertainty as to whether our findings have implications for the coming decades of global warming or only for the longer term. For example, if a decline in tropical Pacific productivity is the main driver of ODZ contraction in a warmer world, then a contraction of the suboxic zones may arise within the coming decades. By contrast, if deep-ocean ventilation is the dominant cause, then our findings would only become important over hundreds of years at the earliest. Regardless, our results indicate that the reported continuing trend towards enhanced open-ocean suboxia^1,2^ may be the result of multidecadal variability^49^ or a transient response to the rapid rate of global warming, and not necessarily a permanent response to a warmer climate.

## Methods

### Core site location

The new FB-δ^15^N analyses were carried out on two sediment cores from the poleward and equatorward margins of the oligotrophic subtropical gyres in the Atlantic (DSDP Site 516) and the Pacific (ODP Site 872), respectively. DSDP Site 516 (Holes Z and F) (30° 17′ S, 35° 17′ W) was drilled at 1,313 m water depth by the Deep Sea Drilling Project (DSDP) during Leg 72 in the southwest Atlantic^50^. The site is located on the Rio Grande Rise. ODP Site 872 (10° 05′ N, 162° 51′ E, 1095 m water depth) was cored from a sea mount around the Marshall Islands in the eastern equatorial Pacific during Ocean Drilling Program (ODP) Leg 144 (ref. ^51^). The unconsolidated sediment consists of abundant, well-preserved planktic foraminifers. Our new data are compiled with previously published FB-δ^15^N data from Pacific ODP Site 1209 and Atlantic ODP Site 1263, most of which are mixed taxa^18^. In addition, mixed-taxa FB-δ^15^N measurements were made in several samples of late Oligocene and early Miocene age from Pacific ODP Site 1211 (32° 0′ N, 157° 51′ E, 2,907 m water depth), which is in close proximity to Site 1209 (Extended Data Fig. 1). Past locations were calculated on the basis of ref. ^52^, or—if available—estimates in ODP reports^53,54^, and are shown in Fig. 1 (map generated using Ocean Data View software^55^ and data from ref. ^55^).

The surface waters above all core sites are environments of complete nitrate consumption and, therefore, should record the δ^15^N of the nitrate supplied from the pycnocline to the surface. Sites 872 and 1209 are located close to the margin of nitrate-bearing surface waters associated with the equatorial Pacific upwelling. This may modify FB-δ^15^N relative to the δ^15^N of the subsurface nitrate supply in the region, most probably increasing FB-δ^15^N (ref. ^56^). As a consequence, any reduction in equatorial upwelling at times in the past might have modestly decreased FB-δ^15^N at Site 872.

We also report new glycerol dialkyl glycerol tetraethers (GDGTs) measurements from DSDP Sites 516 and 588 (26° 07′ S, 161° 14′ E, 1,548 m water depth) as well as ODP Sites 667 (4° 34′ N, 21° 55′ W, 3,536 m water depth), 704 (46° 52′ S, 7° 25′ E, 2,543 m water depth), 730 (17° 44′ N, 57° 42′ E, 1,071 m water depth), 754 (30° 56′ S, 93° 34′ E, 1,247 m water depth), 1146 (19° 27′ N, 116° 16′ E, 2,091 m water depth) and 1263 (28° 32′ S, 2° 46′ E, 2,717 m water depth). The new measurements allow us to generate a continuous record of the SST evolution of low-to-middle and high latitudes across the Cenozoic based exclusively on the TEX_86_ palaeothermometer^21^.

### Age models

**Extended Data Fig. 5:**
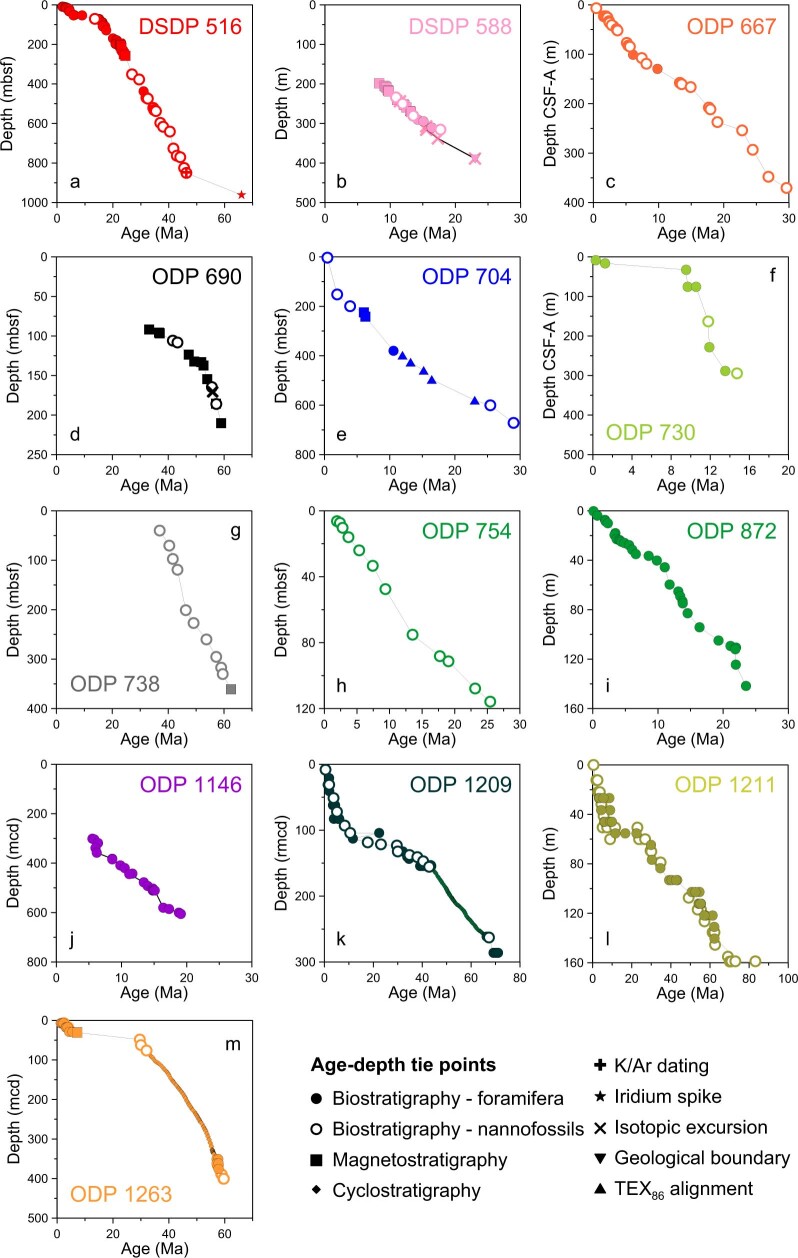
GTS12-adjusted age models with age tie points. **a**, DSDP Site 516 (refs. ^57–59,61–63^). **b**, DSDP Site 588 (ref. ^68^). **c**, ODP Site 667 (ref. ^69^). **d**, ODP Site 690 (ref. ^41^). **e**, ODP Site 704 (ref. ^70^). **f**, ODP Site 730 (ref. ^73^). **g**, ODP Site 738 (ref. ^41^). **h**, ODP Site 754 (ref. ^74^). **i**, ODP Site 872 (ref. ^65^). **j**, ODP Site 1146 (ref. ^75^). **k**, ODP Site 1209 (refs. ^18,66^). **l**, ODP Site 1211 (ref. ^67^). **m**, ODP Site 1263 (refs. ^8,18^).

For DSDP Site 516, we used a combined age model based on biostratigraphic datums^57–59^ adapted to GTS12 (ref. ^60^), magnetostratigraphy by Florindo et al.^61^ and age tie points based on K–Ar dating^62^ and an iridium spike for the K–Pg boundary^63^. For ODP Site 872, we used the age model based on biostratigraphic datums^64,65^ adapted to the GTS12 timescale^60^. The age models for ODP Sites 1209 and 1263 based on biostratigraphy are described in Kast et al.^18^. We revised these age models to the astronomically tuned chronologies recently published by Westerhold et al.^8,66^. The age model for ODP Site 1211 is based on biostratigraphy^67^. The age model for DSDP Site 588 is based on compiled biostratigraphy, magnetostratigraphy and isotope stratigraphy (Pagani et al.^68^ and references therein). Ages for ODP Site 667 were determined by linear interpolation of absolute constraints from biostratigraphic (calcareous nannofossils and foraminifers) datums^69^. Ages for ODP Site 704 were calculated by interpolating biostratigraphic (calcareous nannofossil and foraminifer) datums and magnetostratigraphic data^70^, and by aligning the new TEX_86_ record to the TEX_86_ records from Sites 1171 and U1318 across the middle Miocene climate transition, which are based on high-resolution benthic δ^18^O stratigraphy^71,72^. Ages for ODP Site 730 were determined by linear interpolation of biostratigraphic (calcareous nannofossil and foraminifer) datums^73^. The age model for ODP Site 754 is based on biostratigraphy (calcareous nannofossils)^74^. The age model for ODP Site 1146 is based on biostratigraphy (foraminifers) reported by Nathan and Leckie^75^. Ages for ODP Sites 690/689 are based on refs. ^41,76^. Ages for ODP Site 1171 are based on ref. ^71^. All the biostratigraphic datums and palaeomagnetic reversals were calibrated to GTS12 (ref. ^60^) (Supplementary Data 3 and Extended Data Fig. 5).

### Nitrogen isotope analysis

Around 600–800 individual foraminifers from the species *Globigerinoides conglobatus*, *Trilobatus sacculifer*, *Orbulina universa* and *Sphaeroidinella dehiscens* were picked (250–400-μm size fraction) from the Miocene, Pliocene and Pleistocene sections of DSDP Site 516 and ODP Site 872 (Extended Data Fig. 1). *Dentoglobigerina altispira* and *D. tripartita* were picked from the >400-μm size fraction. In the Oligocene and Eocene sections of Site 516, we measured genus-specific N isotopes on the genera *Dentoglobigerina*, *Turborotalia*, *Acarinina* and *Subbotina*, as well as mixed foraminifers. The FB-δ^15^N measurements were performed in the Martínez-García lab at the Max Planck Institute for Chemistry (MPIC). We used the persulfate oxidation-denitrifier method for FB-δ^15^N first described for planktic foraminifers by Ren et al.^17^, with minor adjustments in the protocol. As a first step, 5–7 mg of foraminifer tests were gently crushed, transferred to a disposable, 15-ml polypropylene, conical-base centrifuge tube and chemically treated to remove external N contamination. 10 ml of Na-polyphosphate solution (pH 8) was added to the sample tubes, which were ultrasonicated for 10 s to remove clays. After rinsing the sample three times with Milli-Q water (by filling and decanting), 7 ml of a dithionite–citric acid solution (100 ml Milli-Q, 6.2 g sodium citrate, 2 g sodium bicarbonate, 5 g sodium dithionite, 400 μl 4N NaOH) was added to each sample^77^. Sample tubes were then placed in a water bath at 80 °C for 30 min. After cooling, all samples were rinsed three times with Milli-Q water. Next, an oxidative cleaning step was performed to remove external organic matter: a potassium persulfate/sodium hydroxide solution (2 g recrystallized potassium persulfate, 2 g NaOH pellets, 100 ml Milli-Q water) was added and autoclaved at 125 °C for 65 min. After cooling, the samples were rinsed four times with Milli-Q water and dried overnight in an oven at 60 °C. Next, 3–5 mg cleaned shell fragments were weighed and dissolved in 45 μl 4N hydrochloric acid to release organic matter for analysis. After the CaCO_3_ dissolution step, organic N was oxidized to nitrate by adding 1 ml of a second recipe of basic potassium persulfate solution (0.7 g recrystallized potassium persulfate, 4 ml 6.25N NaOH solution, 96 ml Milli-Q water). Nitrate concentration was measured for each sample by conversion to nitric oxide followed by chemiluminescence detection^78^. Subsequently, 5 nmol N of nitrate in the sample solution was converted to nitrous oxide using the denitrifier method, and its δ^15^N was measured by gas chromatography–isotope ratio mass spectrometry^79–82^. In contrast to previous studies^19,29,83,84^, samples were not pH adjusted before injection into the denitrifying bacteria; rather, they were injected without adjustment into 2.75 ml media buffered at pH 6.3 and containing the denitrifying bacteria (see ref. ^82,85^ for details). To quantify the precision and accuracy of the corrected isotope values, for each series of 30 samples, a total of nine different in-house (MPIC) foraminifer and coral laboratory standards were analysed. The following were analysed (each in triplicate): a coral standard from the taxon *Porites* (PO-1) with δ^15^N of 6.2 ± 0.3‰, a coral standard from the taxon *Lophelia* with δ^15^N of 10.1 ± 0.4‰ (ref. ^85^) and a mixed foraminifer standard (63–315-μm size fraction) from the North Atlantic (MSM58-17-1) with δ^15^N of 5.84‰ (uncertainty not yet assessed). After calibration with international nitrate isotopic references IAEA-NO3 and USGS-34 and correction for the oxidation blank, the long-term analytical precision for the foraminifer standard was 0.23‰ (1 standard deviation, *n* = 22). The oxidation blank per oxidized sample was typically below 0.2 nmol N.

### Analysis of GDGTs

5–20 g of freeze-dried sediment per sample was extracted and separated into two fractions at the Martínez-García lab at the MPIC, following the protocol proposed by Auderset et al.^86^. After accelerated solvent extraction of the freeze-dried sediment samples, we added 60 μl of an internal standard (C_46_-GDGT, synthesized by Patwardhan and Thompson^87^) for quantification. The extracts were dried in a centrifugal Rocket Evaporator (Genevac) and filtered with a polytetrafluoroethylene filter (0.2-μm pore size) with a 1.4% mixture of hexane:isopropanol (hex:IPA). GDGTs were analysed using a high-performance liquid chromatographer (Agilent, 1260 Infinity) coupled to a single-quadrupole mass spectrometer detector (Agilent, 6130) following the protocol proposed by Hopmans et al.^88^, with some small modifications. Normal-phase separation was achieved with one ultra-high-performance liquid chromatographer silica column (BEH HILIC column, 2.1 mm × 150 mm, 1.7 μm; Waters) maintained at 30 °C. The flow rate of the 1.4% hex:IPA mobile phase was 0.2 ml min^−1^ and kept constant for the first 25 min, followed by a gradient to 3.5% hex:IPA in 25 min and a column-cleaning step with 10% IPA in hexane. We used a single-ion monitoring of the masses *m*/*z* = 1,302.3, 1,300.3, 1,298.3, 1,296.3, 1,292.3, 744.0, 1,050.0, 1,036.0, 1,022.0, 1,020.0 and 1,018.0. The injection volume was 5 μl or 20 μl, depending on the concentration of the samples analysed.

### Pacific and Atlantic FB-δ^15^N stacks

Owing to the emergence and extinction of planktic foraminifer taxa over time, it is important to evaluate the relationship of FB-δ^15^N of extinct foraminifers to that of modern taxa for which the incorporation of the N isotope signal has been studied^19,89,90^. Our new dataset from DSDP Site 516 demonstrates that the FB-δ^15^N of *T. sacculifer*, a modern photosymbiotic, surface-dwelling species, overlaps with the FB-δ^15^N of the extinct species *S. dehiscens*, as well as with the FB-δ^15^N of mixed planktic foraminifers during the early Miocene and late Oligocene (Extended Data Fig. 1). In addition, during the Oligocene and Eocene epochs, the FB-δ^15^N from the genera *Dentoglobigerina*, *Turborotalia* and *Acarinina* overlap well with those of *S. dehiscens* and with mixed species, indicating that there are no notable δ^15^N offsets between modern and extinct species of foraminifera. At ODP Site 872, *O. universa* and *T. sacculifer*, two modern photosymbiotic surface dwellers, show very similar values over the past 15 Myr. In addition, our data show that the FB*-*δ^15^N of these two modern species overlap with that of co-occurring *D. altispira* through the Miocene and Pliocene epochs, despite substantial (but consistent) differences in their N content. *Dentoglobigerina altispira* δ^15^N values, in turn, are similar to those of *D. tripartita* in the early Miocene. In this way, we can combine measurements from different foraminifer species/genera to obtain a continuous record of FB-δ^15^N throughout the Cenozoic (Extended Data Fig. 1). The FB-δ^15^N Atlantic and Pacific stacks presented in Fig. 2c are averages of the species-specific, genus-specific and mixed-taxa values shown in Extended Data Fig. 1. As discussed below, ODP Site 1209 migrated north-westward into the North Pacific subtropical gyre after 40 Myr (Fig. 1), so the ODP Site 1209 record is truncated at this age.

### Core site migration and FB-δ^15^N

Seafloor migration is a ubiquitous concern in Cenozoic-long palaeoceanographic reconstructions. Site 1209 warrants consideration in this regard, as it migrated from the ODZ-influenced central tropical North Pacific into the western North Pacific subtropical gyre between the early and mid-Cenozoic (Fig. 1). Several observations indicate that the sharp decline in FB-δ^15^N at the EECO is not due to site migration. First, the FB-δ^15^N decline into the EECO also occurs in both the North and the South Atlantic, where site migration would not have had such an effect^18^ (Fig. 1). Second, the FB-δ^15^N declines are of similar magnitude for the EECO and the MMCO, arguing that the same dynamic is at work for both warm events. Third, FB-δ^15^N at Site 1209 shows changes that are opposite to the sense expected from site migration: an approximately 6‰ δ^15^N increase from the late Cretaceous to the Palaeocene and an approximately 3‰ increase at around 40 Ma (ref. ^18^). However, the sensitivity of Site 1209 to the isotopic signal of denitrification in the eastern tropical Pacific was eventually reduced by its migration, with the change occurring between the middle Eocene and the early Miocene. This is indicated by the similarity of early Miocene FB-δ^15^N at Site 1211 (a close neighbour of Site 1209) to the FB-δ^15^N at South Atlantic Site 516, both of which are substantially lower than FB-δ^15^N at Pacific Site 872 during the early Miocene (Extended Data Fig. 1). This motivates our use of Site 872 from the Oligocene forward.

### Latitudinal SST stacks

For the presented latitudinal SST stack, we combined a total of 653 new TEX_86_-SST measurements for low (ODP Sites 667 and 730) middle (DSDP Sites 516 and 588, ODP Sites 754, 1146 and 1263) and high latitudes (ODP Site 704) and integrated the records with existing TEX_86_-SST datasets (which include 4,474 data points out of 53 sites and 48 publications) covering the past 69 Ma (refs. ^18,72,91–136^). The data and references are summarized in Supplementary Data 2 and shown in Extended Data Figs. 2 and 3. We categorized the different datasets based on age-adjusted palaeolatitudes using paleolatitude.org^52^. The categories as follows: high latitudes (above 50° N/S), low latitudes (0–20° N/S), mid latitudes (20–40° N/S), transition (40–50° N/S) and costal upwelling regions (Extended Data Fig. 3). Unfortunately, low-latitude sites are scarce and do not provide a continuous SST record across the Cenozoic. Middle-latitude records are more abundant and show SST trends and absolute values that are very similar to those from low latitudes in the intervals in which both records overlap. Consequently, we combined the middle/low latitudes SST records to calculate the latitudinal SST gradient. In Fig. 2, we estimate the gradient between high and middle/low latitudes (ΔSST_L-H_ = SST_mid/low_ − SST_high_) after applying a local regression (LOESS) with a smoothing factor of 0.02 and a 0.2-Myr sampling step (using Python code available on GitHub: https://github.com/audersea/Auderset_etal_2022_nature). Sediment cores located in transition regions (with palaeolatitude between 40° and 50° N/S), as well as upwelling-sensitive locations (ODP 1085, ODP 1087 and ODP 846), were not considered in these calculations to minimize the effect of local changes in upwelling intensity and frontal migrations.

To estimate SST from TEX_86_ values, we used the calibrations proposed by Kim et al.^137^ (TEX^H^_86_) and Tierney and Tingley^138^ (TEX_86 Bayspar_, with the following parameters: prior_mean = 28; prior_std = 10; tol = 0.15; n_samp = 2,500). Overall, the trends obtained when calculating the latitudinal temperature gradient are the same for both calibrations, but there are slightly larger amplitudes of change in the Bayspar-derived gradient (Extended Data Fig. 4b). In addition, we have also estimated the latitudinal gradient using the raw TEX_86_ values for our compilation and compared it to our δ^15^N gradient (Extended Data Fig. 4c). The good correlation between the raw TEX_86_ gradient and the δ^15^N gradient indicate that this is a robust feature that is independent of the calibration used. Finally, we compare our reconstructed latitudinal SST gradient with the one proposed by Cramwinckel et al.^99^ for the time interval between 58 and 30 Ma, based on the combination of benthic oxygen isotopes records for high latitudes and TEX_86_ reconstructions for low latitudes (Extended Data Fig. 4c). Both reconstructions agree well; however, the approach followed by Cramwinckel et al.^99^ is only suitable for periods in which there were no substantial contributions of continental ice to the benthic δ^18^O signal, limiting its application after the Eocene–Oligocene boundary.

Several indices have been proposed to evaluate potential changes in the source of isoprenoid GDGTs over time^110,139–141^. We report the values for these indices in Supplementary Data 2. As suggested by previous studies, we exclude GDGTs data with a high methane index (above 0.4) and high GDGT_RS_ (above 30) from our calculation of the latitudinal temperature gradient (355 samples) (Extended Data Fig. 3a). The branched and isoprenoid tetraether (BIT) index has been proposed to estimate the relative contribution of soil-derived GDGTs to aquatic sedimentary environments characterized by large inputs from nearby soils (that is, costal marine sediments and lake sediments)^142^. In these environments, BIT values below 0.4 have been suggested to indicate minor inputs of terrestrial GDGTs and, therefore, a negligible influence of terrestrial GDGTs on the estimated upper-ocean temperature^139^. Samples with BIT values above 0.4 are typically not used for palaeotemperature calculation owing to the potential bias that soil GDGTs inputs may introduce on the estimated SST. However, the applicability of this index to estimate terrestrial inputs of GDGTs in open-ocean settings has been questioned because (1) branched GDGTs can be produced in situ^143–147^ and (2) degradation rates of crenarchaeol are two times higher than those of branched GDGTs^148^. Thus, in some environments, the BIT index can be high (above 0.4), despite the relatively low terrestrial inputs of brGDGTs. In these cases, samples with BIT > 0.4 have been used for palaeotemperature reconstructions^71,117,129^. In recent years, it has been shown that in situ-produced brGDGTs tend to be characterized by higher abundances of cyclic brGDGTs, resulting in high values for the #rings_tetra_ index, and it has been suggested that #rings_tetra_ values >0.7 are indicative of predominant in situ production of branched GDGTs^149^. Some of the new samples reported here, as well as some of the samples published in previous studies that are included in our compilation, have BIT values >0.4. However, some of the samples with BIT values >0.4 also have values for #rings_tetra_ index >0.7, indicating that the brGDGTs were produced in situ, rather than transported from terrestrial soils. In the calculation of the latitudinal SST gradient, we exclude samples with BIT > 0.4 if the #rings_tetra_ was <0.7 (178 samples) to avoid potential influences of terrestrial GDGTs on the estimated SST (see Extended Data Fig. 3a, Supplementary Data 2 and code at https://github.com/audersea/paper_2022_nature). However, we note that there are no substantial differences in the SST gradient if all the samples with BIT > 0.4 are included in the calculation (Supplementary Data 2), suggesting that the potential influence of terrestrial GDGTs on the estimated SST is probably minimal despite the high BIT values.

## Online content

Any methods, additional references, Nature Research reporting summaries, source data, extended data, supplementary information, acknowledgements, peer review information; details of author contributions and competing interests; and statements of data and code availability are available at 10.1038/s41586-022-05017-0.

## Supplementary information

Supplementary Data 1 FB-δ15N and FB-N content data. Sheets titled ‘DSDP 516’, ‘ODP 872’ contain all species-specific, genus-specific and mixed-taxa foraminifer-bound δ15N and FB-N content measurements from DSDP Site 516 and ODP Site 872, respectively, plotted in Figs. 2 and 3 and Extended Data Fig. 1. Sheet titled ‘ODP 1211’ contains mixed-taxa FB-δ15N and FB-N content measurements from ODP Site 1211, plotted in Extended Data Fig. 1. Sheets titled ‘ODP 1209’ and ‘ODP 1263’ contain genus-specific and mixed-taxa FB-δ15N and FB-N content measurements from ODP Sites 1209 and 1263, respectively, measured by ref. ^18^ with revised age models, plotted in Figs. 2 and 3 and Extended Data Fig. 1. The sheet titled ‘d15N gradient’ contains the calculated nitrogen isotope gradient between the Pacific and the Atlantic plotted in Figs. 2 and 3 and Extended Data Fig. 4.

Supplementary Data 2 TEX_86_ data measured in this study (ODP/DSDP Sites 516, 588, 667, 704, 730, 754, 1146 and 1263) and compilation of all published marine TEX_86_ records. This table contains all GDGTs measurements, calculated indices, temperatures and gradients.

Supplementary Data 3 Applied age models for ODP/DSDP Sites 516, 588, 667, 690, 704, 730, 738, 754, 1146, 1209, 1211 and 1263 with biostratigraphic (calcareous nannoplankton and foraminifer) and magnetostratigraphic datums adjusted to the GTS12 timescale.

## Data Availability

The datasets are available in Supplementary Data 1–3 and the PANGAEA database (10.1594/PANGAEA.943130).

## Code availability

The code used for filtering, smoothing and gradient calculations for the SST and δ^15^N datasets is available on GitHub (https://github.com/audersea/Auderset_etal_2022_nature).
